# The kidney drug transporter OAT1 regulates gut microbiome–dependent host metabolism

**DOI:** 10.1172/jci.insight.160437

**Published:** 2023-01-24

**Authors:** Jeffry C. Granados, Vladimir Ermakov, Koustav Maity, David R. Vera, Geoffrey Chang, Sanjay K. Nigam

**Affiliations:** 1 Department of Bioengineering,; 2 Department of Biology,; 3 Skaggs School of Pharmacy and Pharmaceutical Sciences,; 4 Department of Radiology,; 5 In Vivo Cancer and Molecular Imaging Program,; 6 Department of Pharmacology, School of Medicine,; 7 Department of Pediatrics, and; 8 Department of Medicine (Nephrology), UCSD, La Jolla, California, USA.

**Keywords:** Metabolism, Nephrology, Transport

## Abstract

Organic anion transporter 1 (OAT1/SLC22A6, NKT) is a multispecific drug transporter in the kidney with numerous substrates, including pharmaceuticals, endogenous metabolites, natural products, and uremic toxins. Here, we show that OAT1 regulates levels of gut microbiome–derived metabolites. We depleted the gut microbiome of *Oat1*-KO and WT mice and performed metabolomics to analyze the effects of genotype (KO versus WT) and microbiome depletion. OAT1 is an in vivo intermediary between the host and the microbes, with 40 of the 162 metabolites dependent on the gut microbiome also impacted by loss of *Oat1*. Chemoinformatic analysis revealed that the altered metabolites (e.g., indoxyl sulfate, p-cresol sulfate, deoxycholate) had more ring structures and sulfate groups. This indicates a pathway from gut microbes to liver phase II metabolism, to renal OAT1–mediated transport. The idea that multiple gut-derived metabolites directly interact with OAT1 was confirmed by in vitro transport and magnetic bead binding assays. We show that gut microbiome–derived metabolites dependent on OAT1 are impacted in a chronic kidney disease (CKD) model and human drug-metabolite interactions. Consistent with the Remote Sensing and Signaling Theory, our results support the view that drug transporters (e.g., OAT1, OAT3, OATP1B1, OATP1B3, MRP2, MRP4, ABCG2) play a central role in regulating gut microbe–dependent metabolism, as well as interorganismal communication between the host and microbiome.

## Introduction

Organic anion transporter 1 (OAT1/SLC22A6, originally described as NKT) is a multispecific drug transporter localized to the basolateral membrane of the kidney proximal tubule. OAT1 is involved in the uptake of multiple classes of drugs (e.g., antibiotics, antivirals, NSAIDs, diuretics), endogenous metabolites, toxins, antioxidants, and natural products from the blood into the proximal tubule cell, where they can then be excreted into the urine by apical efflux transporters, reintroduced to the bloodstream, or metabolized by the cell (1–10). While OAT1 favors the transport of organic anions, it can also handle several structurally different small molecules, including some cations and zwitterions (11). To date, the overwhelming majority of research interest in OAT1 has been related to its role in the clearance of drugs, as the US Food and Drug Administration (FDA) and other global regulatory agencies have recommended that novel drug entities be tested for OAT1 interaction due to potential drug-drug interactions (DDIs) occurring at the site of the transporter (12, 13). Despite the pharmaceutical relevance of this transporter, recent studies have highlighted a further role of OAT1 and other drug transporters in endogenous metabolism in the context of several observations (14–17). Many of the metabolites that have received clinical attention— such as 4-ethylphenyl sulfate, p-cresol sulfate, and indoxyl sulfate — are organic anions and well-known OAT1 substrates and have been associated with the gut microbiome.

The gut microbiome plays an important role in the endogenous host metabolism by producing several important metabolites. While these metabolites are generated within the host, they are often the products of complex interactions between the host and the commensal microbes residing in the gut. Gut microbiome–derived metabolites include short-chain fatty acids, secondary bile acids, aromatic amino acid derivatives, polyamines, and several others (18–20). The full repertoire of gut microbiome–derived metabolites and how they are generated remains incomplete, but it is clear that these metabolites play important signaling roles in both healthy and disease states (21). For example, gut bacterial metabolites have been shown to influence the immune system (22). Furthermore, inflammatory bowel disease (IBD), cardiovascular disease, and chronic kidney disease (CKD) are associated with microbial dysbiosis, which can lead to abnormal levels of serum metabolites (23–27). CKD, in particular, is associated with increases in circulating gut-derived uremic toxins due to diminished glomerular and tubular renal function (28–30). OAT1 is known to be critical for the transport of many of these metabolites into the proximal tubule. These gut microbiome–derived metabolites include indoxyl sulfate, p-cresol sulfate, hippurate, and other metabolites and signaling molecules that are organic anions, suggesting that there may be an important role for OAT1 in the mediation of host-microbiome interaction (3, 28, 31). Considering the wide array of substrates transported by OAT1, the competition between drugs, toxins, and gut-derived metabolites at the site of the transporter could also lead to drug-metabolite interactions (DMI) —especially in patients with CKD, who are likely to be taking multiple drugs to treat symptoms associated with comorbidities.

The Remote Sensing and Signaling Theory (RSST) addresses how OAT1, along with other solute carrier (SLC) and ATP-binding cassette (ABC) “drug” transporters, plays a major role in homeostasis of many small molecules, including rate-limiting metabolites, signaling molecules, antioxidants, gut microbe–derived products, vitamins, and cofactors (32). These other SLC and ABC transporters include OAT3 (SLC22A8), organic anion transporting polypeptide 1B1 (OATP1B1, SLCO1B1), OATP1B3 (SLCO1B3), multidrug resistance protein 2 (MRP2, ABCC2), MRP4 (ABCC4), and ABC G subfamily 2 (ABCG2, BCRP). The RSST proposes a complex adaptive system of drug transporters, drug metabolizing enzymes, nuclear receptors, and kinases that regulates endogenous metabolism through transport, metabolism, and conjugation of small molecules with signaling roles between remote organs (e.g., gut, liver, kidney, brain) and multiorganismal systems (e.g., gut microbe–host, mother-fetus) (33, 34). Drug transporters in the SLC22, SLC organic (SLCO), and ATP-binding cassette C subfamily (ABCC) families and their multispecificity (ability to handle structurally different organic anions) are central to the RSST and have been identified as important hubs in a cross-tissue coexpression network, suggesting a principal role in endogenous metabolism at multiple scales (organism to organ to organelle) (14, 32, 35). The RSST has mainly been explored through the lens of interorgan communication (e.g., organ crosstalk) — but an important, understudied aspect is interorganismal communication between the host and the bacterial species in the gut, which is possibly mediated by the role of kidney OAT1 and other drug transporters in modulating gut microbiome host interactions (36).

While OAT1 and OAT3 in the kidney are hypothesized to be central to interorganismal communication via gut-derived metabolites, the hepatic transporters, OATP1B1 and OATP1B3, along with various ABC transporters, such as ABCC2, ABCC4, and ABCG2, are also thought to contribute to the transport of gut microbiome derived compounds (37, 38). Furthermore, drug-metabolizing enzymes are central to the metabolism and conjugation of these compounds along the gut-liver-kidney axis (39–41). Nevertheless, much of the data supporting these notions are from in vitro rather than in vivo studies.

**Figure 1:**
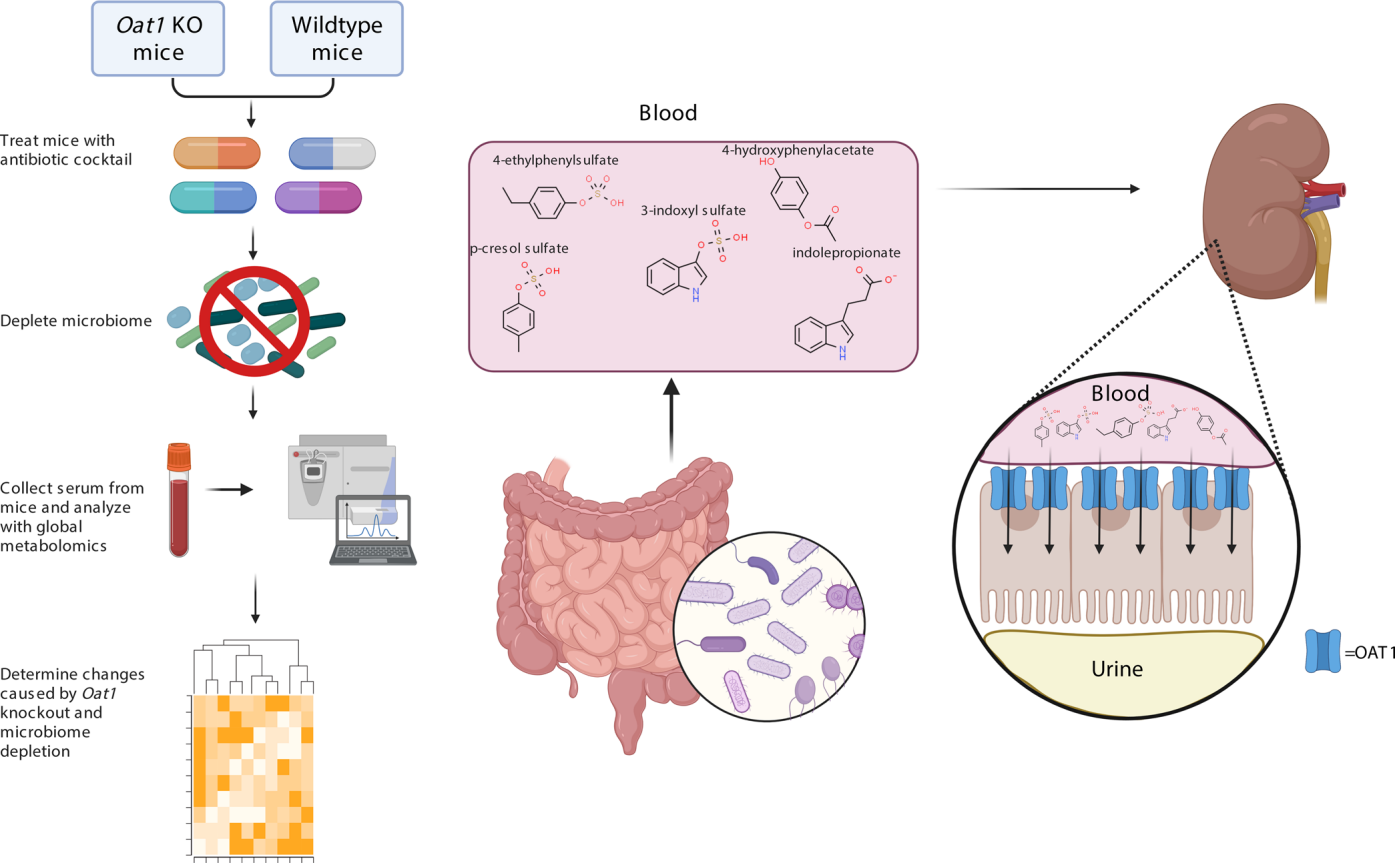
Workflow of experiment and schematic of gut-derived metabolite transport by OAT1. *Oat1-*KO and WT mice were treated with an antibiotic cocktail to deplete the gut microbes. We then assessed the resulting changes on the serum metabolome and determined that many metabolites produced by commensal bacteria in the gut enter the blood stream, where their systemic levels are regulated in vivo by OAT1 in the kidney.

In this work, we focused on the in vivo role of a single multispecific kidney “drug” transporter, OAT1, in the regulation of gut-derived metabolites and the metabolic pathways involving these molecules. We first established the efficacy of the *Oat1-*KO mouse model by demonstrating in vivo alterations in the handling of a well-studied OAT1 substrate, as evidenced by changes in levels in the blood and urine. We then focused on depleting the gut microbiome of these mice and their WT counterparts. Gnotobiotic mice have frequently been used as a model to understand the impact of gut microbes, but while they are generally healthy, they are difficult to compare with WT mice due to differences in their genetic backgrounds, as well as issues related to development, immune defects, and energy metabolism (42, 43). This is a key concern in the context of OAT1, since OAT1 is an α-ketoglutarate antiporter and is, thus, directly linked to aerobic metabolism, upon which the kidney proximal tubule almost exclusively depends (31, 44). Therefore, like many in the field, we chose to employ antibiotic treatment to deplete the gut microbes (45). We then assessed the impact of loss of the *Oat1* gene (*Oat1-*KO versus WT) and microbiome depletion on biochemical pathways, and we applied chemoinformatics approaches to characterize the altered metabolites. To support our in vivo findings, we performed in vitro transport assays and employed a magnetic bead binding assay to evaluate mechanistic relationships between gut-derived metabolites and OAT1. Furthermore, we established clinical and disease relevance of our results by showing that the gut microbiome–derived metabolites that are OAT1 dependent are significantly affected in a clear example of human DMI and in a rodent CKD model. Our results indicate that OAT1 plays a surprisingly important role in the handling of a number of gut-derived metabolites and, consistent with the RSST, mediates interorganismal communication between the host and gut microbes, in large part by regulating the circulating levels of these compounds (Figure 1).

## Results

### Clearance of OAT1-interacting compound altered in vivo in KO mice.

We first characterized our *Oat1-*KO mice and their WT counterparts by measuring the levels of Tc-99m mercaptoacetyl-triglycine (MAG3) in the urine (bladder) and the blood. Tc-99m MAG3 is a probe compound used in the assessment of renal function that is nearly entirely eliminated by tubular secretion. Previous studies have demonstrated that Tc-99m MAG3 is a rat OAT1 (rOAT1) substrate in vitro and that its uptake is inhibited by classic OAT1 inhibitors, such as PAH and probenecid (46). Clinically, results in humans have shown that MAG3 levels in the blood were elevated following treatment with PAH and probenecid (47). These observations were supported by assays using HEK293 cells expressing human OAT1, which showed that the protein is involved in the uptake of Tc-99m MAG3 (47).

**Figure 2:**
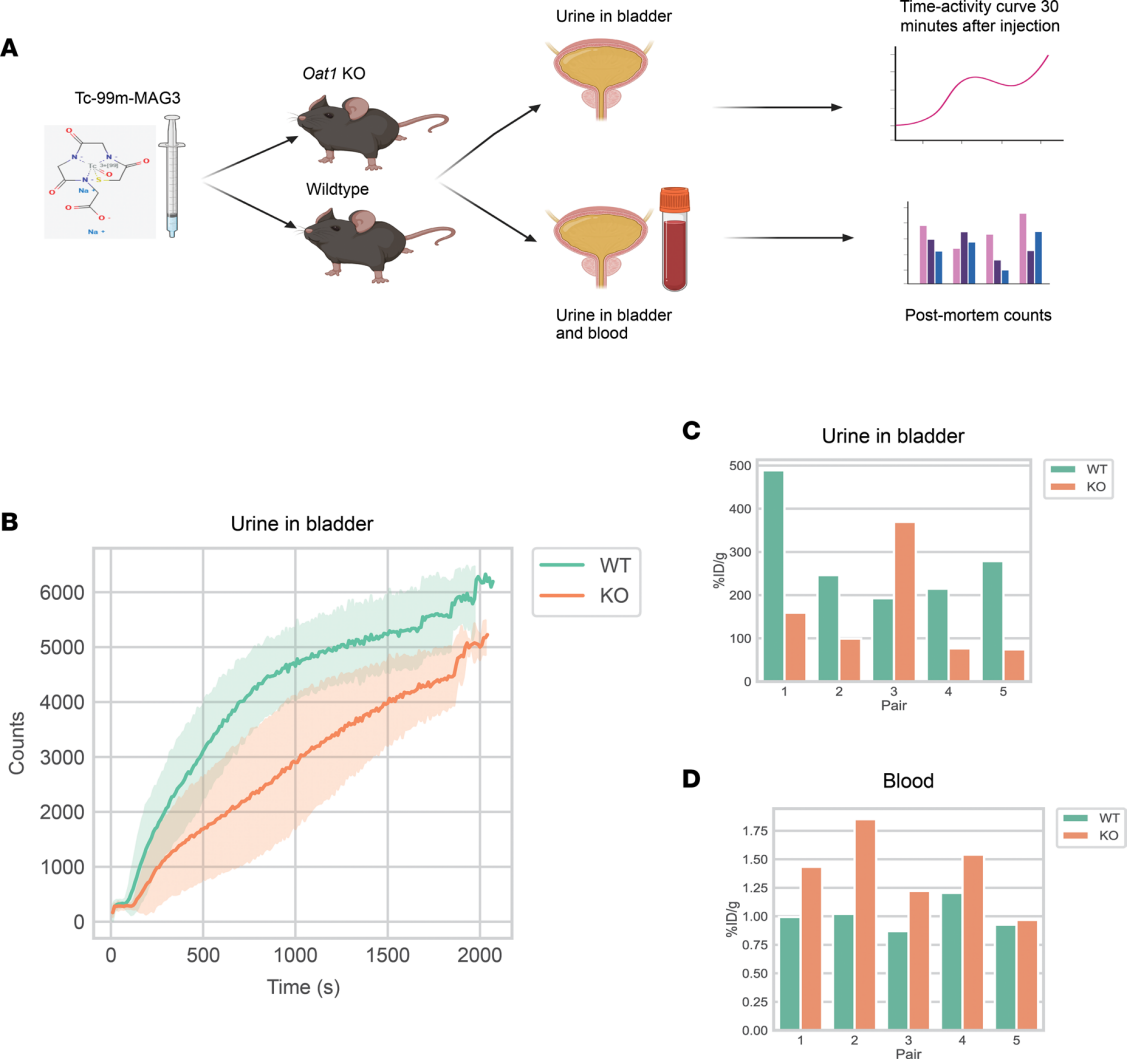
Tc-99m MAG3, an OAT1 substrate, has different clearance patterns in WT and KO mice. (**A**) Schematic for measurement of Tc-99m MAG3 in the urine in the bladder and the blood. (**B**) Over the course of 30 minutes, the bladders of WT mice (*n* = 5) reached their maximal levels of Tc-99m MAG3 more quickly than KO mice (*n* = 5). The central line represents the mean, while the error bands represent a SD at each time point. (**C**) In weight-scaled postmortem γ counts, 4 of 5 pairs of mice showed higher levels of Tc-99m MAG3. Given that OAT1-mediated transport is often the rate-limiting step for clearance into the urine, this pattern demonstrates the functional usefulness of genetic KO for the studies that follow. (**D**) In weight-scaled postmortem gamma counts, Tc-99m MAG3 levels in the blood were higher in the *Oat1-*KO mice in 4 of 5 pairs. Since OAT1 is expressed at the basolateral membrane of the proximal tubule, it follows that blood levels of a substrate would be elevated in the *Oat1*-KO mice. %ID/g: percent injected dose over gram.

We evaluated whether the *Oat1-*KO and WT mice had different clearance patterns with this well-established OAT1 substrate (Figure 2A). Tc-99m MAG3 was administered to the mice via tail-vein injection, and its levels were monitored over the course of 30 minutes using a γ camera. We mainly focused on the bladder, as Tc-99m MAG3 quickly passes through the kidneys into the urine. We found that the WT bladders reached their maximal levels of Tc-99m MAG3 more quickly than the *Oat1-*KO mice following direct injection of the probe compound (Figure 2B). These results were further supported by postmortem γ counts scaled to weight, which showed that, for 4 of 5 pairs, the bladder levels of Tc-99m MAG3 were higher in the WT mice (Figure 2C) and the blood levels were higher in the KO mice (Figure 2D). Given that OAT1 is considered the rate-limiting step for excretion of many organic anions into the urine, our results support the usefulness of the KO mice as in vivo models for analyzing OAT1-related function.

### Gut microbiome was depleted in Oat1-KO and WT mice.

**Figure 3:**
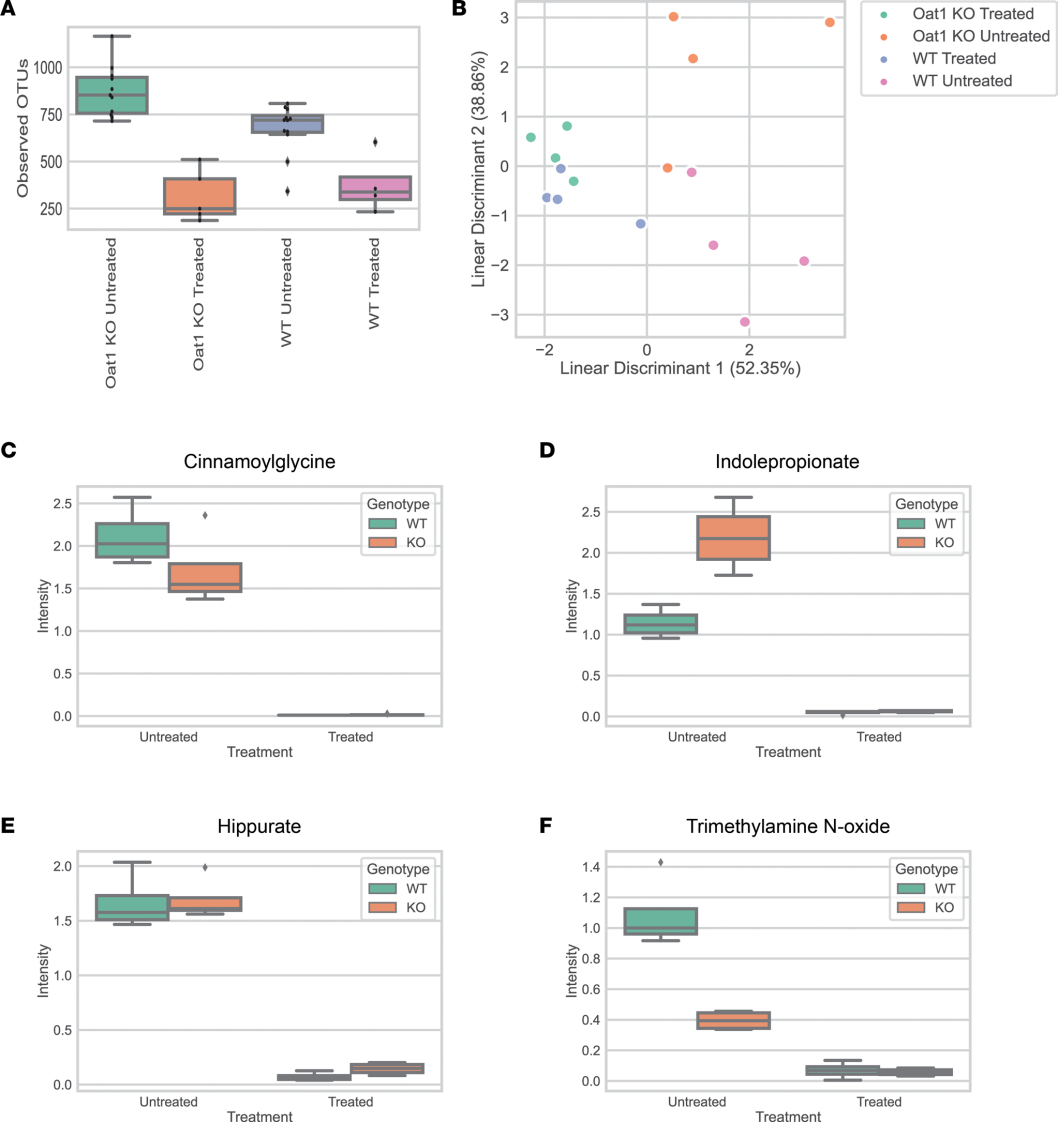
An antibiotic cocktail depleted the gut microbes in *Oat1-*KO and WT mice and decreased the circulating levels of gut-derived metabolites. (**A**) Metagenomic analysis of mice showed that observed OTUs are decreased in mice (*n* = 12 for WT untreated, *n* = 11 for *Oat1-*KO untreated, *n* = 5 for *Oat1-*KO treated, *n* = 4 for WT treated) treated with antibiotic cocktail. (**B**) Linear discriminant analysis revealed separation between the metabolomic profile of the 4 groups (*n* = 4 for all groups). (**C**–**F**) The serum abundance of well-established gut-derived metabolites (cinnamoylglycine [2.31×10^-10^], indolepropionate [5.66×10^-08^], hippurate [7.99×10^-08^], and trimethylamine N-oxide [3.85×10^-04^] with different origins is significantly decreased in treated groups, as determined by corrected 2-way ANOVA. (*n* = 4 for all groups). Box plots include the median as the central line, the lower quartile as the lower limit of the box, the upper quartile as the upper limit of the box, the max value as the upper limit of the whisker, and the minimum value as the lower limit of the whisker. Diamonds indicate a value that falls outside of the interquartile range.

Previous in vivo and in vitro experiments have shown that OAT1 has several putative gut-derived substrates, such as indoxyl sulfate, p-cresol sulfate, and hippurate (17, 31, 48). While these metabolites are useful in understanding a potential role for OAT1 in regulating circulating levels of gut microbe–derived metabolites, the results were collated from multiple past experiments performed under a variety of conditions and not designed to evaluate the in vivo contribution of gut microbiome and renal OAT1 to host systemic metabolism. In this work, we depleted the gut microbiome in both WT and KO mice through the administration of an antibiotic cocktail (ampicillin, vancomycin, neomycin, and metronidazole [AVNM]). The AVNM antibiotic cocktail has been established as an effective method of depleting the gut microbiome and — in contrast to germ-free mice, which can develop metabolic problems that can lead to obesity (43) — seemed less likely to confound the essential role of OAT1 in kidney aerobic metabolism (31, 44) and the tendency to hepatic steatosis seen in approximately 24 month old *Oat1*-KO mice (16). The AVNM cocktail was administered through a vehicle control in the drinking water for 4 weeks (45). Following the administration of the cocktail, depletion of the gut microbiome was confirmed via metagenomic analysis of the feces, which showed a significant decrease in the number of operational taxonomic units (OTUs) for AVNM-treated mouse groups (Figure 3A). The global metabolic profiles of all animals were separable by linear discriminant analysis (Figure 3B), and several well-established gut-derived metabolites were significantly decreased in the serum of AVNM-treated animals, regardless of genetic background (Figure 3, C–F). Furthermore, quantitative PCR (qPCR) using 16S primers for *Eubacteria* also showed a significant decrease in gut microbes (Supplemental Figure 1; supplemental material available online with this article; https://doi.org/10.1172/jci.insight.160437DS1).

### Loss of Oat1 and microbiome depletion significantly affect the levels of over 200 metabolites.

**Figure 4:**
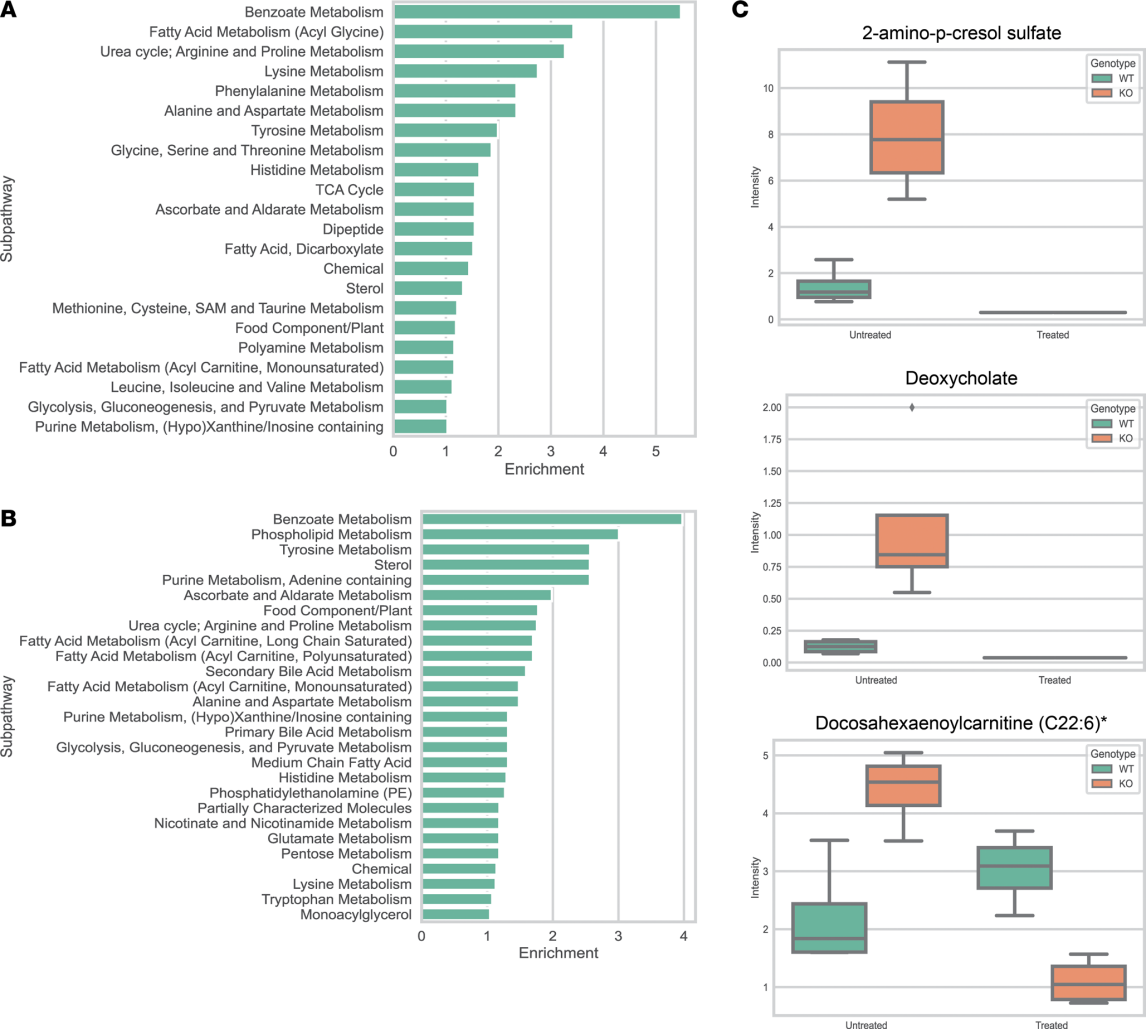
Genetic KO of *Oat1* and antibiotic treatment lead to multiple altered biochemical pathways. (**A**) Enrichment results from the 103 metabolites significantly altered by loss of *Oat1* show that Benzoate Metabolism, Fatty Acid Metabolism, and others are among the most affected subpathways. (**B**) Enrichment results from the 162 metabolites significantly altered by microbiome depletion reveal that Benzoate Metabolism, Phospholipid Metabolism, Tyrosine Metabolism, and others are among the most affected subpathways. (**C**) 2-Amino–p-cresol sulfate, deoxycholate, and docosahexaenoylcarnitine (C22:6)* were significantly affected by the interaction between genotype and treatment, as determined by 2-way ANOVA. Asterisk denotes the identity has not yet been confirmed based on a chemical standard, but there is high confidence in its identity. Box plots include the median as the central line, the lower quartile as the lower limit of the box, the upper quartile as the upper limit of the box, the max value as the upper limit of the whisker, and the minimum value as the lower limit of the whisker.

Since OAT1 is localized to the basolateral (blood-facing) side of the proximal tubule, its absence directly affects the circulating levels of metabolites in the serum. To identify these compounds, we performed a 2-way ANOVA to determine the individual impact of genotype (*Oat1-*KO versus WT), where a metabolite was considered altered if it had an FDR-corrected *P* value below 0.05. Although metabolomics of the *Oat1*-KO has been previously performed, this is the first time to our knowledge that nearly 1,000 compounds were measured, and here — apart from including the effects of microbiome depletion — we also focus on all altered metabolites, not just elevated metabolites (3, 16, 17, 31, 49). Global metabolic profiling detected a total of 964 metabolites in the volume-adjusted serum samples collected from these mice. Based on the Metabolon grouping of these metabolites, this analysis covered 10 biochemical superpathways (e.g., Lipid, Amino Acid, Xenobiotic) and 109 biochemical subpathways (e.g., Primary Bile Acid Metabolism, Tryptophan Metabolism, Benzoate Metabolism), with each metabolite belonging to 1 superpathway and 1 subpathway. We identified 103 significantly altered metabolites due to the absence of OAT1 (Supplemental Table 1), including several metabolites that are known to directly interact with OAT1, such as pyridoxate, indoxyl sulfate, and p-cresol sulfate (50). We then performed an enrichment analysis of these metabolites and found that over 20 subpathways were altered, with enrichment values of 1 or greater, indicating an outsized effect. Benzoate Metabolism and Fatty Acid Metabolism (Acyl Glycine) were among the most significantly affected pathways (Figure 4A).

Having established depletion of the gut microbiome with AVNM treatment (by decreased OTUs and qPCR), we then analyzed how this impacted the serum metabolome of the microbiome-depleted mice. We found that 162 metabolites were significantly altered in the serum of the microbiome-depleted mice (Supplemental Table 2). Thus, microbe depletion has a direct impact on over 100 metabolites, including several metabolites that have previously been established as gut derived, like cinnamoylglycine, indolepropionate, and others (51). Subpathway analysis revealed that Benzoate Metabolism, Phospholipid Metabolism, and Tyrosine Metabolism were among the most altered subpathways, and over 20 subpathways had enrichment values of 1 or greater (Figure 4B). Given that there is no current consensus on the full range of commensal gut bacteria–derived metabolites that enter the host circulation, we interpreted these metabolites to be products of gut microbiome–associated metabolism with the understanding that, for some metabolites, the levels in the serum may be due to complex interactions between bacterial species themselves and, upon entry into the host circulation, indirect effects on host metabolic pathways that likely include complex feedback and/or feedforward loops, with secondary bile acid metabolism being a good example (52).

### Deoxycholate levels depends on an interaction between OAT1 and the gut microbiome.

We then explored the statistical interaction between the 2 independent variables: loss of *Oat1* and microbiome depletion. Only 3 metabolites (2-amino–p-cresol sulfate, deoxycholate, docosahexaenoylcarnitine [C22:6]) were impacted by the interaction between the variables, which implies that genotype and treatment, together, influence few metabolites compared with the individual effects of genotype versus treatment (Figure 4C). C22:6 and 2-amino–p-cresol sulfate are poorly characterized, but deoxycholate has a well-established signaling role, suggesting an important role for OAT1, together with gut microbes, in regulation of this important bile acid signaling molecule (53–55).

### Pathways and chemoinformatics analyses of the 40 metabolites affected by both loss of Oat1 and microbiome depletion.

**Figure 5:**
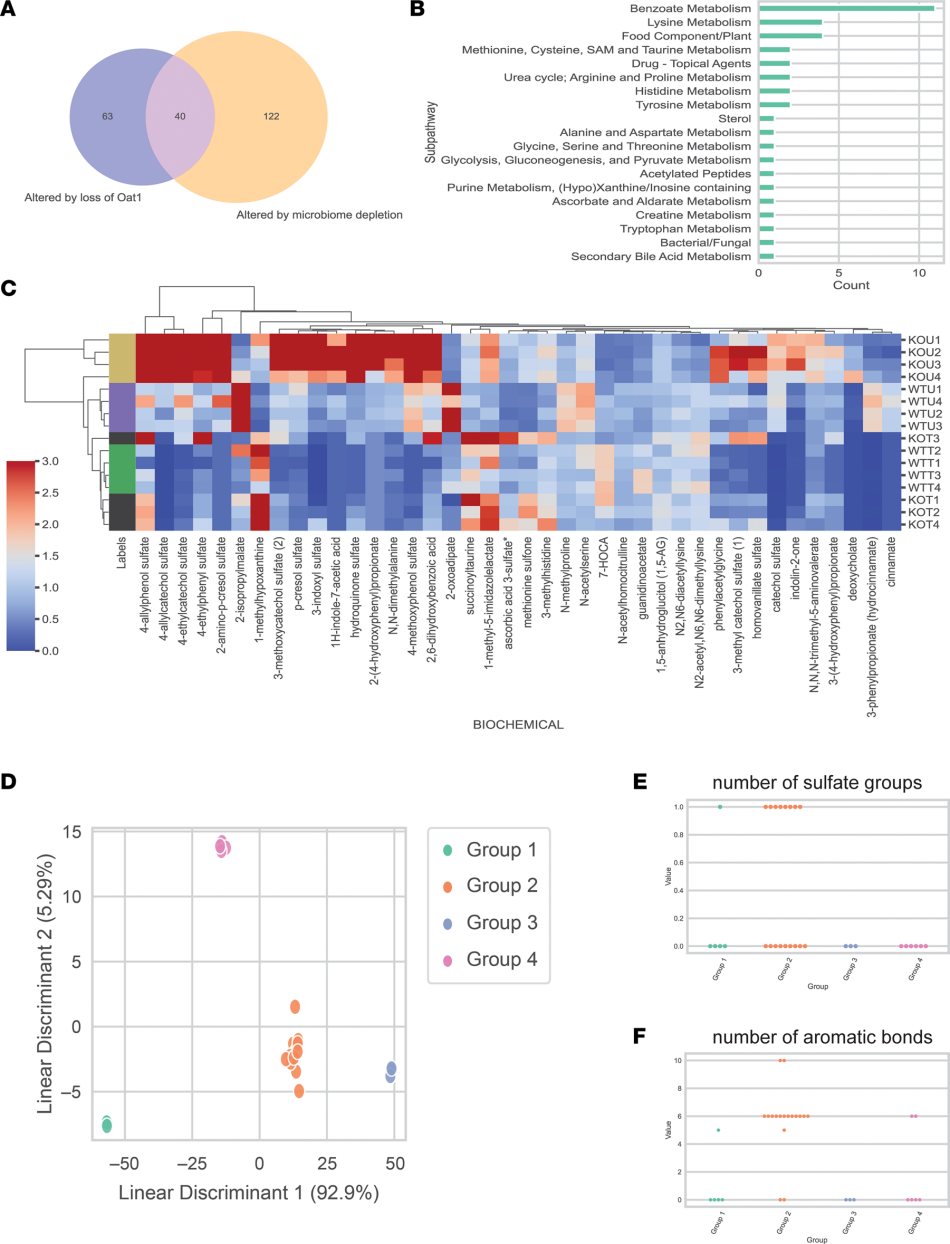
Treatment and genotype overlap in their impact on 40 circulating metabolites. (**A**) Of the 103 metabolites affected by loss of *Oat1* and the 162 metabolites affected by microbiome depletion, 40 metabolites overlapped. (**B**) The 40 metabolites belonged to 19 unique subpathways, with 11 belonging to the Benzoate Metabolism subpathway. (**C**) The levels of the 40 metabolites are shown, with the scaled intensity of each metabolite being scaled to range from 0 to 3 for improved visualization, though there are some metabolites that have higher scaled intensities. The individual groups of mice are shown on the *y* axis, and the individual metabolites are shown on the *x* axis. KOU, *Oat1-*KO untreated; KOT, *Oat1-*KO treated; WTU, WT untreated; WTT, WT treated. (**D**) Linear discriminant analysis (LDA) shows clear separation between the 4 groups of metabolites altered by both loss of *Oat1* and microbiome depletion. Thirty-one of the 40 overlapping metabolites have available chemical structures (Group 1, 5 metabolites with structures; Group 2, 17 metabolites with structures; Group 3, 3 metabolites with structures; Group 4, 6 metabolites with structures). We were unable to find clearcut information for 2-amino–p-cresol sulfate, N2-acetyl, N6,N6-dimethyllysine, 4-allylcatechol sulfate, 4-ethylcatechol sulfate, 4-methoxyphenol sulfate, N-acetylhomocitrulline, succinoyltaurine, 3-methoxycatechol sulfate (2), or 1-methyl-5-imidazolelactate. (**E**) Thirteen of the 22 compounds in Group 2 (elevated by KO and decreased by microbiome depletion) were sulfated. Only 1 other compound in the other groups was sulfated. (**F**) Number of aromatic bonds was one of the features that most separates the 4 groups of compounds affected by *Oat1* KO and microbiome depletion. Group 2 had higher numbers of aromatic bonds than the other 3 groups.

We then aimed to identify the overlap between the 103 metabolites altered by loss of *Oat1* and the 162 metabolites affected by microbiome depletion. We found 40 metabolites (Figure 5A) that satisfied both criteria and found that some subpathways, particularly Benzoate Metabolism with 11 compounds and Food Component/Plant with 5 compounds, were markedly affected in the overlap (Figure 5B). These 40 compounds could be separated into 4 distinct groups: Group 1 (elevated by loss of *Oat1* and elevated by microbiome depletion); Group 2 (elevated by loss of *Oat1* and decreased by microbiome depletion); Group 3 (decreased by loss of *Oat1* and elevated by microbiome depletion); and Group 4 (decreased by loss of *Oat1* and decreased by microbiome depletion) (Figure 5C).

The metabolites we were most interested in were those that were in Group 2 (elevated in the *Oat1*-KO mice and decreased due to microbiome depletion), as these are likely OAT1 substrates that are generated by the gut microbiome. Of the 40 metabolites, 22 fell into this group, including indoxyl sulfate and p-cresol sulfate. We were also interested in the 9 metabolites in Group 1 (increased in the *Oat1*-KO mice and increased due to microbiome depletion), as microbiome depletion can also lead to increases in specific metabolites by reducing the species that metabolize these compounds. Finally, the last 2 groups were more difficult to interpret from the OAT1 perspective, as there is no clear renal physiological mechanism for their decreases in circulation; nonetheless, there were 7 metabolites in Group 3 (decreased in the *Oat1-*KO mice and decreased by microbiome depletion) and 5 metabolites in Group 4 (decreased in the *Oat1-*KO mice and increased by microbiome depletion). We then aimed to structurally characterize the 31 metabolites with known chemical structures.

Chemoinformatics analyses can shed light on sets of molecular properties that help define particular groups of metabolites altered by a biological experiment. In this case, for example, we were most interested in Group 2 (the metabolites that were both elevated due to loss of OAT1 and decreased after microbiome depletion), as these were not only the largest group, but also most likely to be gut microbe–derived organic anion metabolites transported by OAT1 in vivo. This could yield a kind of “signature” of metabolites that originate in the gut microbiome and then follow the gut-kidney or gut-liver-kidney axes to OAT1 in the renal proximal tubule cells.

To this end, we first calculated molecular properties for the 783 compounds with valid chemical structures. We performed linear discriminant analysis of the 31 compounds (Group 1, 5 metabolites with structures; Group 2, 17 metabolites with structures; Group 3, 3 metabolites with structures; Group 4, 6 metabolites with structures) that had available chemical structures and observed clear separation between the groups (Figure 5D). We then analyzed the weights of the top 2 linear discriminant analysis axes, which together explained over 95% of the variance. Among the most influential variables were number of sulfate groups and number of aromatic bonds (Figure 5, E and F). Again, we were most interested in the 17 compounds with chemical structures that were elevated by loss of *Oat1* and decreased by microbiome depletion (Group 2), and these compounds tended to have a higher number of aromatic bonds. The gut microbiome is known to handle a number of aromatic compounds, such as tryptophan and tyrosine derivatives. With respect to sulfation, 8 of the 17 metabolites in Group 2 featured a sulfate group. This was especially interesting because only 1 sulfated metabolite was present in the other 3 groups combined (Figure 5F). Nevertheless, this is consistent with the notion that certain diet-derived compounds are metabolized by the gut microbiome before being “tagged” via sulfation by the host for excretion, primarily through the urine (56, 57).

### Gut-derived metabolites interact with human OAT1 in cell-based transport assays.

**Figure 6:**
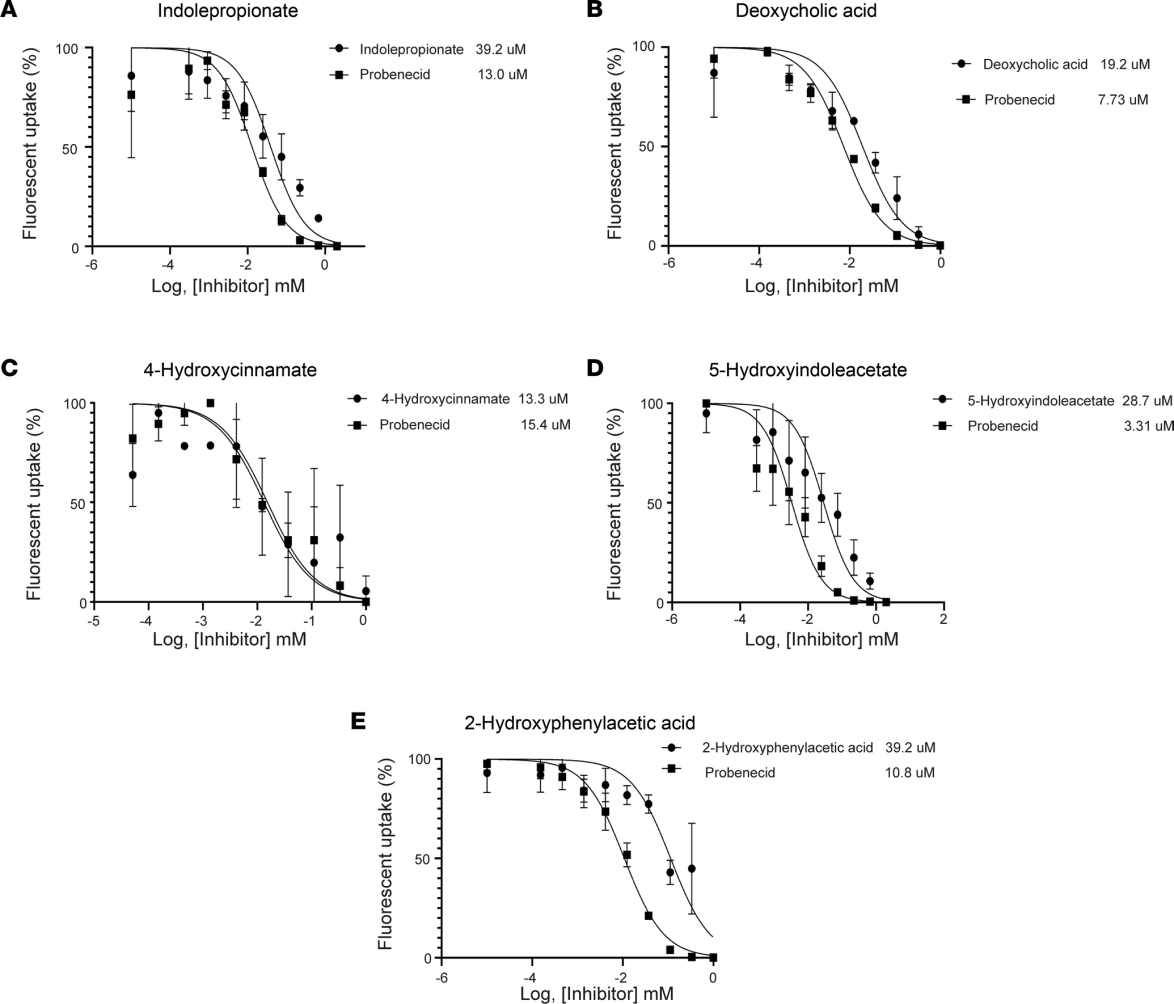
OAT1 transport is inhibited by gut-derived compounds in vitro. (**A**–**E**) Indolepropionate (*n* = 4), deoxycholic acid (*n* = 4), 4-hydroxycinnamate (*n* = 2), 5-hydroxyindoleacetate (*n* = 5), and -hydroxyphenylacetic acid (*n* = 2) inhibited the transport of 6-carboxyfluorescein in OAT1-expressing HEK293 cells. Controls were performed with the prototypical OAT1 inhibitor probenecid, and IC_50_ values from at least *n* = 2 assays are shown.

While the *Oat1-*KO mouse model is critical for establishing potential in vivo OAT1 substrates, the complex physiology in vivo could lead to alterations caused by a factor other than loss of OAT1 function. To evaluate a mechanistic interaction between metabolites and OAT1, we performed in vitro cell-based transport assays. Competitive inhibition assays were carried out for deoxycholate, indolepropionate, 4-hydroxycinnamate, 2-hydroxyphenylacetic acid, and 5-hydroxyindoleacetate, which are all thought to be gut-derived metabolites. In the statistical analysis of the serum metabolome, deoxycholate was affected by genotype-treatment interaction, 2-hydroxyphenylacetic acid was affected by genotype, and indolepropionate was affected by treatment. Although 4-hydroxycinnamate and 5-hydroxyindoleacetate were not impacted by either variable, for experimental evaluation, they were included because they are derived from cinnamate and indole, respectively. Each metabolite showed comparable inhibition when OAT1 was treated with probenecid, the prototypical inhibitor of OAT1 activity. The relatively low IC_50_ values (<115 μM) suggest that these metabolites interact with OAT1 with high affinity (Figure 6).

### A magnetic bead binding assay shows direct physical OAT1 interaction with gut-derived metabolites.

**Figure 7:**
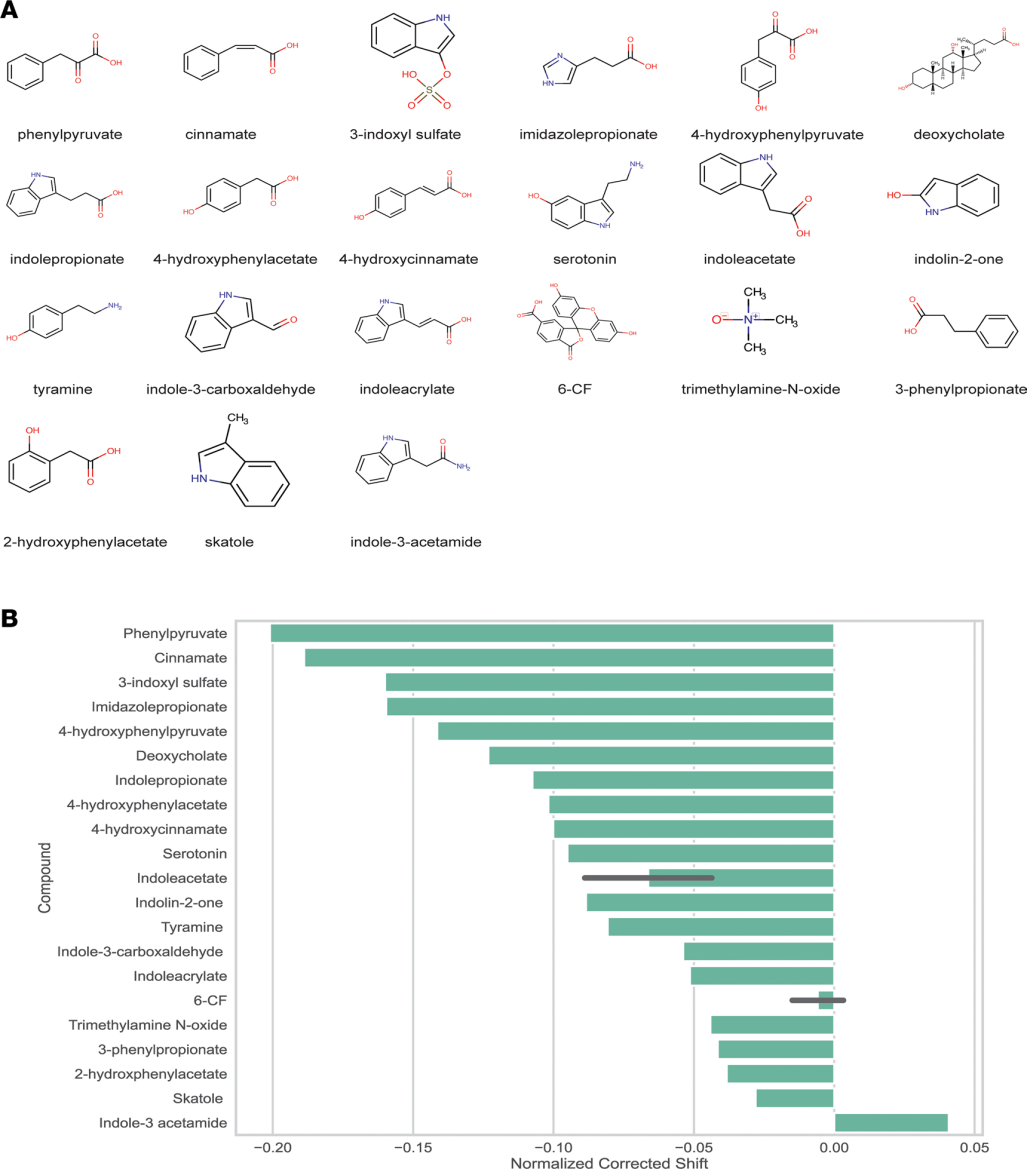
OAT1 binds gut-derived compounds in vitro. (**A**) Twenty gut-derived metabolites and a control compound (6-carboxyfluorescein) were measured using the magnetic bead binding assay. (**B**) All but 5 of the compounds (15 of 20) showed a substantial normalized corrected shift in 6-carboxyflourescein signal, indicating that these compounds bind to OAT1.

To further evaluate our results, we also employed a potentially novel magnetic bead binding assay using 6-carboxyfluorescein to analyze 20 gut-derived metabolites, a number of which were measured in the serum metabolomics (Figure 7A). The strength of this is that the assay requires relatively low amounts of OAT1 protein compared with those used for other methods (e.g., fluorescence polarization technique). Using this method, we surveyed metabolites mainly known to derive from tryptophan and tyrosine, some of which were measured in our in vivo experiments and have been evaluated in transport assays (Table 1). Overall, we found that 15 of the metabolites resulted in a significant shift, further supporting a direct interaction between the gut microbe–derived metabolites and OAT1(Figure 7B). Taken together with the cell-based in vitro transport assays that also support a direct interaction of the gut-derived metabolites with OAT1, as well as the fact that many of the interacting metabolites were from Group 2 (elevated in *Oat1*-KO mice and decreased by microbiome depletion), the data support the in vivo involvement of OAT1 in the regulation of this group of gut microbe–derived metabolites.

### The in vivo OAT1-dependent, gut microbe–derived metabolites overlap with those impacted by a CKD model (5/6 nephrectomy).

We then aimed to contextualize our findings by comparing our results to metabolomics data previously generated by our group in a rodent 5/6 nephrectomy model of CKD (28). This model is thought to capture aspects of progressive CKD, and renal capacity is dramatically reduced over time (28). In these experiments, plasma was collected from animals who had undergone a 5/6 nephrectomy and their healthy controls, and the relative levels of hundreds of metabolites were measured. In the comparison between the nephrectomized and healthy animals, many of the elevated metabolites have been shown to include numerous uremic solutes or uremic toxins (28). However, the nature of their diminished clearance remains unclear, since this model of renal insufficiency impacts both tubular and glomerular function. By comparing the metabolites elevated in the 5/6 nephrectomy with the 40 metabolites that are gut derived and altered in the *Oat1-*KO mice, we were able to identify metabolites that are likely impacted by diminished proximal tubule function, as that is where OAT1 is primarily expressed. Thus, 7 metabolites (indoxyl sulfate, p-cresol sulfate, phenylacetylglycine, 4-ethylphenyl sulfate, 3-methylhistidine, N-acetylserine, 2-isopropylmalate) are likely uremic solutes or uremic toxins transported by OAT1 and produced by the gut microbiome (Table 2).

### Gut-derived metabolites transported by OAT1 are involved in human DMI.

**Figure 8:**
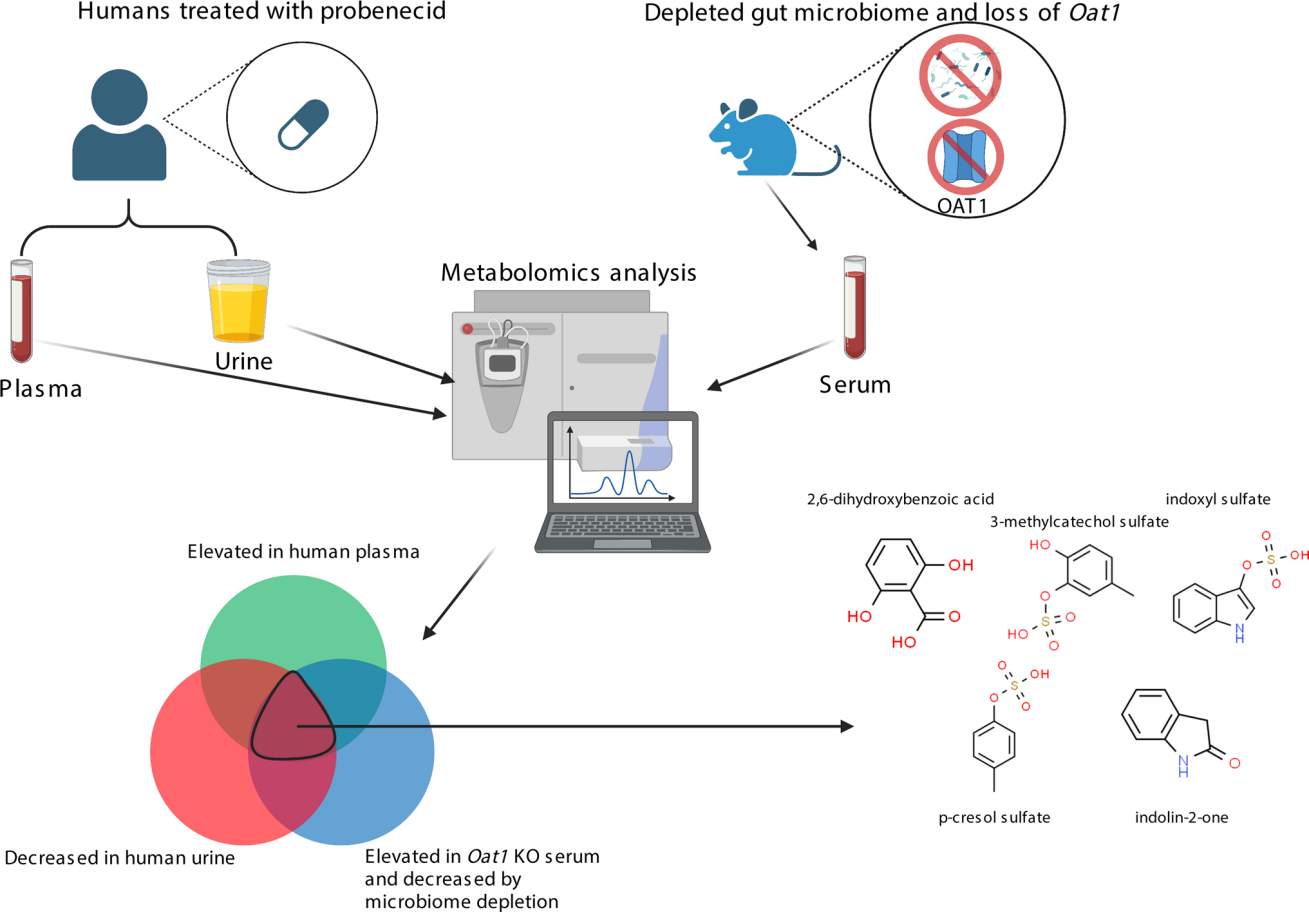
Gut-derived metabolites mediated by OAT1 kidney function are involved in clinical drug–metabolite interactions. Of the 40 metabolites significantly affected by both loss of *Oat1* and microbiome depletion (1), 8 were also implicated in clinical drug–metabolite interactions with the drug probenecid. These compounds were both elevated in the plasma and decreased in the urine, indicating that OAT1-mediated movement is the rate-limiting step. Of the 8 metabolites, 5 had chemical structures and are shown in the figure. The remaining 3 are 4-ethylcatechol sulfate, 4-methoxyphenol sulfate, and 4-allylcatechol sulfate. There were also 20 of 40 metabolites decreased in the urine, and there were 11 of 40 increased in the plasma.

Having established that 40 metabolites are likely OAT1 mediated and produced by the gut microbiome in a mouse model, we then aimed to understand the clinical relevance of these results. A recent metabolomics study by our group analyzed the plasma and urine of healthy volunteers before and after probenecid treatment and identified dozens of unique short-term DMI (1). While that study did not concern itself with gut microbe–derived metabolites, we were able to reanalyze that data in the context of the new data in this study. Thus, we performed an overlap of the metabolites implicated in the present *Oat1*-KO microbe-depleted mouse study with those significantly elevated in the plasma and significantly decreased in the urine of humans treated with probenecid (Figure 8, Supplemental Table 3, and ref. 1). Over a quarter of the OAT1-transported gut-derived metabolites from the current mouse study (11 of 40) were significantly elevated in the plasma of the probenecid treated humans, including indoxyl sulfate, p-cresol sulfate, and other compounds. When we compared the 40 metabolites with those significantly decreased in the urine of probenecid treated humans, we found that half the metabolites (20 of 40) were present in both lists. Interestingly, 4-ethylphenyl sulfate, a compound associated with autism, was present in this overlap, along with others (58). When all 3 lists were overlapped, 8 metabolites were present, with most being sulfated organic anions (Figure 8). These gut microbiome–derived metabolites are dependent on OAT1 and are also involved in DMI.

## Discussion

The loss of *Oat1* and the depletion of the gut microbiome (Figure 3), separately and together, have major effects on systemic metabolism (Figure 4 and Supplemental Tables 1 and 2). The genetic KO of *Oat1* primarily leads to elevated metabolites, presumably because they are no longer able to enter the proximal tubule and must remain in the blood, whereas the depletion of the gut microbiome mainly leads to lower circulating levels of gut-derived metabolites by eliminating the species that synthesize or modify the compounds. Both conditions together — KO of *Oat1* and gut microbe depletion — provide perhaps the deepest glimpse to date of how the renal organic anion transport system works together with the gut microbiome to regulate systemic levels of many well-known metabolites and signaling molecules (Figure 1).

Most impressive were the effects of gut microbiome depletion on metabolites elevated due to loss of *Oat1* (compared with the WT with a normal microbiome). Many of these metabolites elevated in the *Oat1*-KO mice were significantly, and sometimes markedly, decreased after gut microbiome depletion (Figure 5). Based on this in vivo data, and the evidence presented here for a direct in vitro interaction between a number of these metabolites and OAT1 in transport and binding assays (Figures 6 and 7), it is highly likely that the largest fraction of these metabolites were derived from the gut microbes and are regulated by OAT1, which is important, given the important roles of these metabolites play in the immune response and other key physiological systems (22).

That said, it should be noted that there were also instances of metabolites significantly decreased by loss of *Oat1*, as well as metabolites significantly increased by microbiome depletion. The interpretation of these changes is less clear but may be due to indirect effects such as elevation in the KO of another metabolite transported by OAT1 that inhibits the synthesis of the decreased metabolite.

Overall, we identified 40 metabolites (31 with chemical structures) (Supplemental Table 3) that were significantly impacted by both loss of *Oat1* and gut microbiome depletion, with 22 of those compounds being elevated due to KO and decreased due to antibiotic treatment. These included derivatives of tryptophan and tyrosine. Chemoinformatics analyses of this group of metabolites (e.g., elevated in the *Oat1*-KO mice and decreased after gut microbiome depletion) revealed that these metabolites tended to have aromatic rings and more sulfate groups, which is generally consistent with known molecular properties of OAT1 substrates (Figure 5) (59, 60). The high fraction of sulfated metabolites is particularly interesting and likely indicative of the interaction of gut-derived metabolites with sulfotransferases in the liver before transport into the kidney proximal tubule by OAT1 — examples, similar to that of indoxyl sulfate, of the conjunction of remote interorganismal communication and organ crosstalk (61). Indeed, 13 of the 22 putative gut-derived OAT1 substrates in Group 2 are sulfated compounds. However, since other OATs, such as OAT3 (62), have a strong preference for steroid sulfates, it may be the context in which the sulfated compounds are presented that determines OAT1 interaction. Our data suggest that a 1- or 2-ringed structure with a sulfate may be preferred by OAT1.

The strongest candidates for in vivo OAT1-transported gut microbiome–derived metabolites would seem to be those that are (a) elevated in the plasma of *Oat1*-KO mouse; (b) decreased by gut microbiome depletion; and (c) shown to directly interact with the transporter. Thus, to further analyze the in vivo metabolomics results, a number of the identified metabolites (and others that have been suggested to be gut derived) were tested in vitro for interaction with human OAT1 overexpressed in cells. These compounds displayed IC_50_ values in transport assays that indicate a strong interaction with the transporter. Additional support was gained from a magnetic bead binding assay demonstrating that a fluorescent prototypical OAT1 substrate was displaced by a number of gut-derived metabolites (Table 1). While the transport assays are traditionally used to determine interaction, the magnetic bead binding assay provides further context for the nature of the interaction and allows for more rapid screening of small molecules.

Our results are also highly relevant to disease and clinical settings. We found that many of the metabolites implicated in our study were elevated in 5/6 nephrectomy rodent models of CKD, indicating that these gut-derived and OAT1-mediated metabolites can be altered in the setting of diminished renal function (28). Among the metabolites present in both studies were indoxyl sulfate, p-cresol sulfate, and 4-ethylphenyl sulfate, further supporting the view that OAT1 and the gut microbiome are jointly involved in the generation/handling of uremic toxins.

We also addressed the important clinical issue of DMI. A previous study by our group analyzed the DMI caused by the drug probenecid (1). While that clinical study did not focus on the gut microbiome, given the results from the present mouse study, we investigated whether DMI with an OAT-inhibiting drug (probenecid) had a major impact on the disposition of gut-derived metabolites. Indeed, 8 metabolites were elevated in the plasma of probenecid-treated humans, decreased in the urine of probenecid-treated humans, and altered by both loss of *Oat1* and microbiome depletion (Figure 8). Furthermore, 20 of the 40 metabolites were decreased in the urine, while 11 of the 40 were elevated in the plasma. OAT1 is a major transporter of antibiotics, antivirals, NSAIDs, diuretics, and other common drugs (63). Our analysis suggests that gut microbiome–dependent DMI at the level of OAT1 could be quite widespread (64). This requires further study.

Taken together, our results indicate that OAT1 is a crucial intermediary between the host and the microbes, with 40 metabolites of the 162 metabolites decreased by gut microbiome depletion being presumably influenced by OAT1. This suggests that as much as 25% of microbiome-influenced metabolism may be modulated by OAT1. However, we must also note that the metabolomics platform we used is biased toward compounds that have already been identified and are likely relevant in clinical or research settings. Consistent with previous work, we too observed dozens of metabolites decreased by microbe depletion that were the products of hepatic metabolism (20, 31).

The communication between the host and the gut microbes is complex, but it is clear that these 2 entities have coevolved over time to develop a symbiotic relationship from the perspective of metabolism. This is evidenced by the gut-derived metabolites, which cannot be produced by the host alone, that have important signaling roles, such as nuclear receptor and G-protein–coupled receptor activation (65, 66). Among the implicated metabolites, deoxycholate, indoxyl sulfate, indolepropionate, and others have been shown to have important signaling roles (67–70). While the signaling roles of gut-derived metabolites with respect to target proteins is an important field of research, our results indicate that much more attention needs to be paid to the proteins that regulate their levels in biofluids, as well as in tissues and along organ axes (e.g., gut-liver-kidney, gut-brain). These proteins, which include OAT1, are important avenues by which the host and its gut microbes interact, as they control the bioavailability of signaling molecules.

SLC22 family members (e.g., OATs, organic cation transporters [OCTs], organic cation and zwitterion transporters [OCTNs]) and other multispecific drug transport proteins (e.g., ABCG2, ABCC2) are hubs in a recently proposed Remote Sensing and Signaling Network (32). The Remote Sensing and Signaling Theory (RSST) emphasizes the importance of the adaptive network of multispecific transporters, enzymes, and nuclear receptors — working together with oligo-specific and monospecific proteins — in the optimization of the levels of numerous metabolites in cells, tissues, organs, and bodily fluids, such as blood, cerebrospinal fluid, and urine (10, 14, 32–34, 41). These proteins have been extensively studied from the pharmaceutical perspective, but their ability to handle structurally diverse molecules is perhaps most important from the perspective of endogenous and gut-derived metabolites. The theory mainly serves as a framework to describe interorgan communication, such as in the gut-liver-kidney axis (where most drug transporters and drug metabolizing enzymes are highly expressed), to maintain and reestablish homeostasis of key metabolites, signaling molecules, antioxidants, and other small molecules with “high informational content” (10). While we focused on the individual role of OAT1 in this work, it is likely that the combined network of transporters and enzymes, including cytochrome P450s (CYPs), sulfotransferases (SULTs), uridine 5’-diphospho-glucuronosyltransferases (UGTs), ABCCs, and SLCOs, also contribute to the handling of gut-derived products. Indeed, it has been shown that the SLCOs transport the gut-derived secondary bile acids (71).

One aspect of the RSST that has received less attention is interorganismal communication between the host and the gut microbes (72). In-depth study is likely to have important clinical ramifications — for instance, in understanding the role of gut microbe–derived uremic toxins in the aberrant metabolism of CKD (28, 30, 61). If we treat the gut microbiome as an independent organ, it can be considered to express thousands of transporters and enzymes, many of which exhibit broad substrate specificity and are commonly involved in movement of metabolites, and the synthesis of small molecules as well as their hydrolysis, reduction, or removal of conjugated groups (73). It is established that the substrates and products of the enzymatic reactions occurring in bacterial species overlap with those of the transporters and enzymes in the host, enabling interorganismal communication via multi-, oligo-, and monospecific enzymes that are part of the Remote Sensing and Signaling Network. Furthermore, there is evidence that the gut microbiome and its metabolites can impact transporter and enzyme expression. In renal disease, it has been shown that gut-derived metabolites have an impact on the expression of several drug-metabolizing enzymes in the kidney (74). The presence of gut microbes has been shown to alter the expression of hepatic DME genes in mice (75). Indoxyl sulfate, an important gut-derived uremic toxin, has also been shown to regulate the level of OAT1 expression through AHR activation and, along with other aspects of this pathway, has been interpreted as an experimentally verified example of the RSST (68, 76).

Establishing that the communication between the host and the gut microbes is so strongly mediated by renal OAT1 opens up many questions. For example, it has been shown that several drugs — including statins and ACE inhibitors — have an impact on the composition of the gut microbiome and, by implication, the levels and composition of gut microbe–derived metabolites in the host. However, the mechanisms are unclear. Since these and other drugs impacting the gut microbiota are OAT1 substrates, it is possible that these changes could alter competition of the drug with metabolites at the level of the transporter. There is now good evidence in humans to support this kind of DMI at the level of OAT1 (1).

In summary, our studies indicate that multispecific transporters and enzymes combine with the gut microbiome to regulate circulating levels of key metabolites, including those with signaling capabilities. Since these effects need not be limited to OAT1 in the kidney, it is worthwhile to perform similar analyses with multispecific transporters (e.g., SLCO and ABCC families) expressed in the kidney, liver, intestine, and other organs. A much more complex and clinically actionable picture of the regulation of microbiome-dependent host metabolism is likely to emerge. This can be particularly useful for studying DMI.

## Methods

### Animals.

Adult male KO and WT mice were housed in a 12-hour light-dark cycle and allowed ad libitum access to food and water. *Oat1*-KO mice were generated and maintained as previously described (49). Feces were collected from mice weekly in sterile 2 mL centrifuge tubes, flash frozen, and stored at –80°C. Animals were sacrificed by CO_2_ inhalation, and blood samples were extracted from mice by cardiac puncture. Serum was extracted, and samples were flash frozen and stored at –80°C until further analysis.

### Tc-99m MAG3 imaging.

Live-imaging experiments were performed with the In Vivo Imaging Shared Resource at the Moore’s Cancer Center at the UCSD. Mice were transported from the vivarium to the In Vivo Imaging Shared Resource. In total, 100 μCi of Tc-99m MAG3 was injected by tail vein into mice prior to imaging. Adult, male KO (*n* = 5) and WT mice (*n* = 5) were imaged in 5 separate pairs containing 1 KO mouse and 1 WT mouse each. Mice were initially weighed and anesthetized (2% isoflurane, 200 mL/min flow of O_2_). Mice were placed on their backs on the high-resolution γ imager (γ imager; BioSpace) fitted with a high-resolution, low-energy collimator. For 30 minutes, time-activity curves were collected from the kidneys, liver, and bladder. Following imaging, blood, urine, kidneys, liver, and spleen were isolated and weighed from each mouse for radioactive assessment using a γ counter.

### Microbiome depletion protocol.

Over a 4-week period, mice were given a 125 mL antibiotic cocktail or vehicle control in place of drinking water. The cocktail consisted of 1 mg/mL of neomycin sulfate (Thermo Fisher Scientific, BP-2669-25), 1 mg/mL of ampicillin (Sigma-Aldrich, A9518-100G), 1 mg/mL of metronidazole (Alfa Aesar, H60258), 0.5 mg/mL of vancomycin (Alfa Aesar, J62790), and 3.75 mg/mL of Kool Aid grape drink powder (Kraft-Heinz Foods Company). The Kool Aid encouraged consumption of the cocktail. Antibiotic cocktails were replenished every other day on Monday, Wednesday, and Friday. New solutions were passed through a 0.22 μm cellulose acetate sterilizing filter (Corning, 430517). Bottles of antibiotic cocktail were also wrapped in foil to prevent light damage. The weights of mice and the amount of antibiotic cocktail consumed were monitored over the treatment period as markers of consumption. Following a 1-week decrease in both weight and consumption, mice returned to near their original weights (Supplemental Figure 1).

### Assessment of gut microbiome depletion.

For 16S variable region sequencing, murine fecal samples from week 0 and week 4 (end of treatment time point) were extracted using the MagMax Microbiome Ultra Nucleic Acid Isolation Kit (Thermo Fisher Scientific, A42357) according to the manufacturer’s instructions. Variable (V4) regions of 16S SSU rRNA were amplified using 515F-806R primers according to the protocol described in http://earthmicrobiome.ucsd.edu/protocols-and-standards/16s/ 16S sequencing was performed by the Institute for Genomic Medicine (IGM) UCSD. Resulting files were analyzed using the web-based Qiita tool (77).

For the qPCR, feces total RNA was extracted using the RNeasy PowerMicrobiome Kit (Qiagen, 26000-50) according to the manufacturer’s instructions. A cDNA library of the RNA extracted was created using the SUPERSCRIPT III kit (Invitrogen, 18080-044) with random hexamers (Invitrogen, 48190011) as primers according to the guidelines of the manufacturer. RNA and cDNA were quantified using a Nanodrop 1000 (Thermo Fisher Scientific, 2353-30-0010) and were subsequently used to load equal amounts of cDNA for the qPCR performed. Each well in the qPCR plate contained 20 ng of cDNA from fecal RNA. Duplicates of each sample containing *Eubacteria* primers were utilized, targeting the universal 16S rRNA gene that captures a majority of bacteria (78). 1.KAPA SYBR FAST Universal kit was utilized with an accompanying protocol (Roche, KK4608).

A default 16S metagenomics workflow was run in Qiita under Qiime version 1.9. Raw reads were demultiplexed and trimmed, and OTUs were closed-reference picked using SortMeRNA v2.1 with a 97% sequence similarity minimum. OTUs were assigned taxonomies from the GreenGenes 16S rRNA database, version 13_8, and tabulated into feature and reference tables. All analyses of the feature and reference tables were performed in the Qiita platform and graphed using the Python package, Seaborn.

### Metabolomics analysis of WT and KO mice (untreated and treated).

Serum samples were shipped on dry ice to Metabolon Inc. for preparation and metabolomics. Per Metabolon Inc., proteins were removed from the serum and 5 fractions were generated for different mass spectrometry methods. Each sample passed quality control compared with well-characterized controls and was analyzed by ultrahigh performance liquid chromatography–tandem mass spectroscopy (UPLC-MS/MS). Peaks were identified and associated with defined compounds based on retention time, mass/charge ratio, and MS/MS spectral data. Quantification of peaks was performed using AUC.

### Physicochemical analysis of metabolites.

Two-dimensional chemical structures were obtained from 783 of the measured metabolites by their Pubchem IDs. Seventy-seven 1-dimensional descriptors were calculated for each metabolite using ICM Molsoft-Pro. These molecular properties were then trimmed to eliminate heavily correlated features using a Spearman’s correlation cutoff of 0.90. Linear discriminant analysis was then applied and visualized using the Python packages Seaborn and scikit-learn. Visualizations of the chemical structures were performed using RDKit.

### In vitro OAT1 transport assays.

Human embryonic kidney (HEK) cells stably overexpressing human OAT1 (SOLVO Biotechnology) were used for in vitro inhibition assays. Cells were maintained in DMEM (Thermo Fisher Scientific, catalog 11965092) supplemented with 10% FBS (Thermo Fisher Scientific, catalog 26140079), 1% penicillin/streptomycin (Thermo Fisher Scientific, catalog 15140122), and blasticidin (InvivoGen, catalog ant-bl-1), a selective marker for OAT1 expression. Cells were tested for mycoplasma contamination, and no contamination was observed. Prior to functional assays, cells were plated in 96-well plates and grown for 24 hours or until confluent in media without blasticidin. Metabolites were added at either 1 mM or 2 mM, and serial dilution was performed down all columns. A fixed concentration of 10 μM 6-carboxyfluorescein (6-CF) was introduced to each well for 10 minutes. Cells were rinsed 3 times in ice-cold PBS, and the fluorescence was measured using a fluorescence plate reader. IC_50_ values were calculated using GraphPad Prism 9. Controls were carried out using probenecid, an established inhibitor of OAT1 function.

### Magnetic bead binding assay.

The OAT1 gene was cloned into a third-generation lentiviral vector system. The full-length protein was expressed as GFP fusion in HEK293 cells to check for protein expression. OAT1 protein was solubilized using n-Dodecyl-β-D-Maltoside (bDDM) detergent and then purified by immobilization on magnetic beads coupled to anti-flag antibody (MedChemExpress, HY-K0207). We used magnetic beads (5 μm) as the basis for a binding assay to screen small molecule compounds competing with a well-established OAT1 substrate, 6-CF, which was used as a fluorescence tracer. Loss of fluorescence when challenged with another substrate at a given concentration indicated potential competition for the same binding site. Multiple compounds and concentrations were assayed in a 96-well format using flow cytometry, gating directly on the scattering of the beads. Candidate compounds were initially screened at 3–10 times the concentration of 6-CF (kept constant at 6 μM, for example).

Fluorescence measurements were conducted using a Novacyte flow cytometer (Agilent) with a sampler that reads 1 sample well at a time at regular time intervals. Since these magnetic beads are denser than water, we used 50% glycerol to reduce bead sedimentation. Loss of fluorescence when challenged with candidate compounds at different concentrations was normalized against the maximum fluorescence due to 6-CF binding to OAT1, which was also measured periodically between every set of 10 samples. These check-point measurements (evaluating 6-CF binding to OAT1, in this case) spaced in time allowed us to monitor and to correct fluorescence due to bead sedimentation over time. Using this approach, we screened 20 compounds and categorized them (as OAT1 binder versus nonbinder) based on their competitive efficiency against 6 μM 6-CF, selecting for loss of fluorescence signal compared with the relative error in repeated measurements of 6 μM 6-CF binding to OAT1 alone.

### 5/6 Nephrectomy model.

In the metabolomics data from 5/6 nephrectomy model previously described by us (28), 1 kidney and 2/3 of the other were removed to model diminished renal function. A sham operation was performed on the healthy controls. After 2 weeks, plasma samples were collected from the animals following sacrifice. The samples were metabolically profiled, and comparisons between 5/6 nephrectomized and healthy, nonnephrectomized animals were analyzed. We then compared these metabolites with those in Supplemental Table 3.

### Human DMI.

As previously described by us, plasma and urine samples were collected from 20 healthy participants before and 5 hours after an oral dose of probenecid (1). These samples were metabolically profiled, and pre- and postcomparisons were analyzed to determine compounds that were significantly altered. We analyzed compounds that were elevated in the plasma, those that were decreased in the urine, and those that satisfied both criteria. We then compared these metabolites to those in Supplemental Table 3.

### Statistics.

Scaled intensity for each metabolite was normalized to volume, and missing values were imputed with the lowest value for the compound. For all fold-change calculations, scaled intensities were averaged and compared with each other. Statistical comparisons were performed using the Python module, statsmodels (https://www.statsmodels.org/stable/index.html) and were made between groups by 2-way ANOVA following log transformation and FDR correction, with *P* < 0.05 being considered significant. Enrichment for each superpathway and subpathway was calculated using the number of total metabolites measured and the number of metabolites in each respective superpathway and subpathway, as previously described (16).

### Study approval.

All experimental protocols were approved by the UCSD IACUC, and the animals were handled in accordance with the Institutional Guidelines on the Use of Live Animals for Research. All the experiments described here follow the ARRIVE (Animal Research: Reporting of In Vivo Experiments) guidelines.

## Author contributions

JCG and SKN wrote the manuscript. JCG, VE, and KM conducted experiments and acquired data. JCG and KM analyzed data. SKN, DRV and GC provided reagents and resources. SKN conceived of the project. SKN and GC designed various aspects of the research studies. All authors reviewed the manuscript.

## Supplementary Material

Supplemental data

Supplemental tables 1-3

## Figures and Tables

**Table 1:**
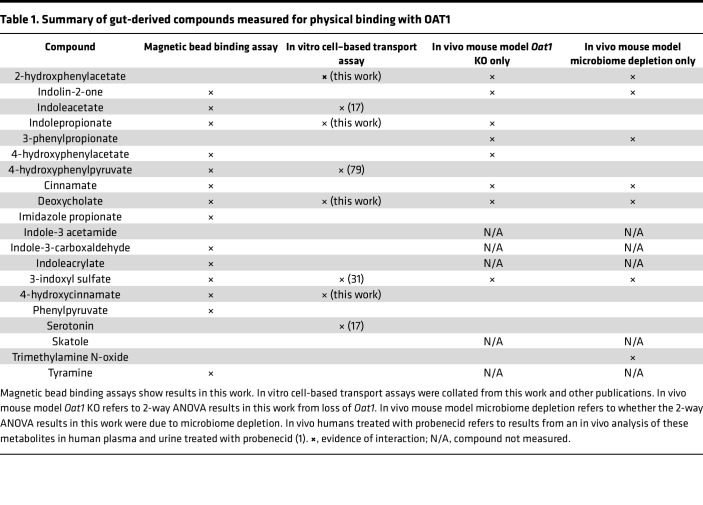
Summary of gut-derived compounds measured for physical binding with OAT1

**Table 2:**
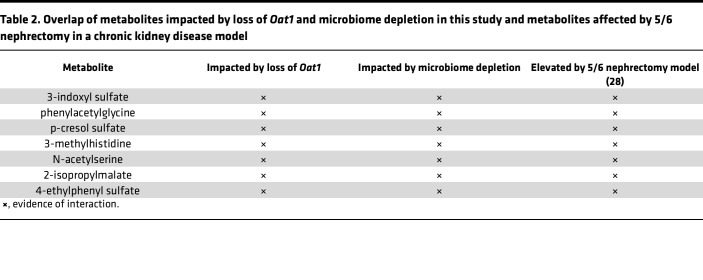
Overlap of metabolites impacted by loss of *Oat1* and microbiome depletion in this study and metabolites affected by 5/6 nephrectomy in a chronic kidney disease model

## Version 1 01/24/2023

Electronic publication
