# Supplementary material for: The kidney drug transporter OAT1 regulates gut microbiome–dependent host metabolism
Source: JCI Insight. 2023 Jan 24;8(2):e160437. doi: 10.1172/jci.insight.160437 (PMC9977316; doi:10.1172/jci.insight.160437)
Supplement: Supplemental data [file jciinsight-8-160437-s066.pdf]

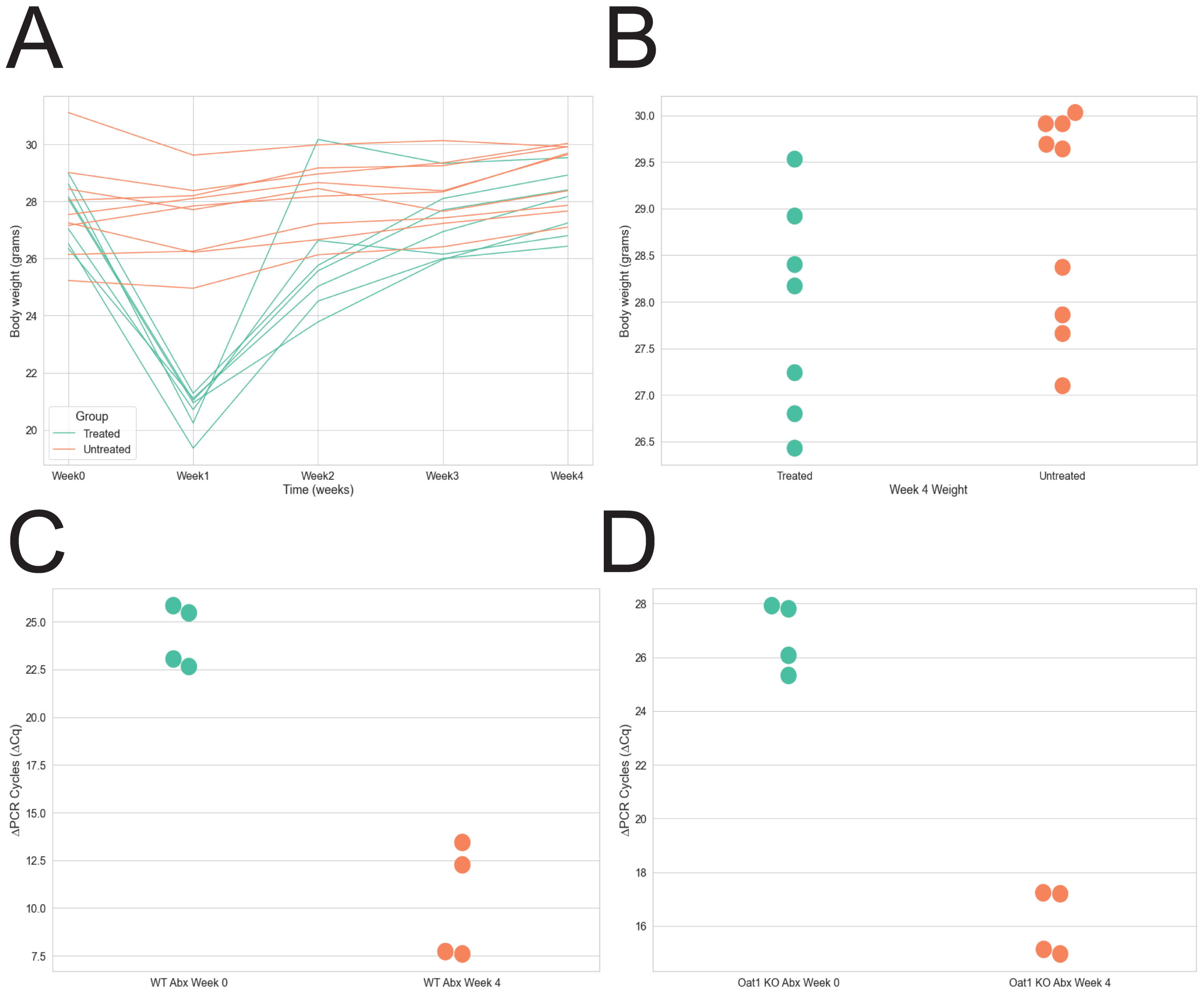

Supplementary Figure S1: Antibiotic treatment leads to depletion of gut microbes. A) All of the treated mice (n=7 for treated mice, n=9 for untreated) initially lost weight before recovering following the first week. B) By the fourth week, the mice had returned to their original weight and there was no significant difference in weight between the AVNM treated and untreated mice using student's t-test. C) The wild type mice had their microbes depleted after four weeks of AVNM treatment (n=4 for Week 0, n = 4 for Week 4). D) The Oat1 KO mice had their microbes depleted after four weeks of AVNM treatment (n=4 for Week 0, n = 4 for Week 4).
